# Effectiveness and safety of lanadelumab in ethnic and racial minority subgroups of patients with hereditary angioedema: results from phase 3 studies

**DOI:** 10.1186/s13223-022-00721-y

**Published:** 2022-09-24

**Authors:** Timothy J. Craig, Rafael H. Zaragoza-Urdaz, H. Henry Li, Ming Yu, Hong Ren, Salomé Juethner, John Anderson, the HELP and HELP OLE Study Investigators

**Affiliations:** 1 Allergy Asthma and Immunology, Departments of Medicine, Pediatrics, Biomedical Sciences, Penn State University, 500 University Drive, Hershey, PA 17033 USA; 2 University of Puerto Rico School of Medicine, UPH, San Juan, Puerto Rico USA; 3 Institute for Asthma and Allergy, Chevy Chase, MD USA; 4 Takeda Pharmaceuticals, Inc., Lexington, MA USA; 5 Cytel Inc., Cambridge, MA USA; 6 Alabama Allergy & Asthma Center, an affiliate of AllerVie Health, Birmingham, AL USA

**Keywords:** Hereditary angioedema, Long-term prophylaxis, Lanadelumab, Race, Ethnicity, Disparity

## Abstract

**Background:**

The COVID-19 pandemic has highlighted disparities in healthcare, particularly in the United States, even though disparities have existed since the organization of the modern healthcare system. Recruitment of patients from racial and ethnic minority groups is often minimal in phase 3 clinical trials, and is further exacerbated in the case of trials for rare diseases such as hereditary angioedema (HAE). This can lead to a gap in the understanding of minority patients’ experiences with these diseases and their response to potential treatment options.

**Methods:**

We reviewed data from phase 3 double-blind (HELP) and open-label extension (HELP OLE) trials of lanadelumab, a monoclonal antibody developed for long-term prophylaxis against attacks of HAE. Efficacy (attack rate reduction) and safety (adverse events) results from White patients were compared descriptively to those from Hispanic/Latino patients, Black/African Americans, and other minority Americans.

**Results:**

Not surprisingly, few minorities were recruited across both studies: 9.5% Black, 2.4% Asian, and 7.1% Hispanic/Latino versus 88.1% White and 91.7% non-Hispanic/non-Latino received lanadelumab in HELP, and 4.7% Black, 0.9% Asian, 0.9% other, and 6.1% Hispanic/Latino versus 93.4% White and 93.4% non-Hispanic/non-Latino were enrolled in HELP OLE. Although these studies were conducted in the United States, Canada, Europe, and Jordan, all minorities were from the United States. Despite the number of minority patients being far less than expected for the population, there was no evidence that either efficacy or adverse event profiles differed between ethnic or racial groups.

**Conclusions:**

The HELP and HELP OLE studies described herein recruited far fewer minorities than would be ideal to represent these populations. However, evidence suggests that the effectiveness and tolerance of lanadelumab are similar between the groups. Nonetheless, the disparity in recruitment into research for minorities has significant room for improvement.

*Trial registration* NCT02586805, registered 26 October 2015, https://clinicaltrials.gov/ct2/show/record/NCT02586805. NCT02741596, registered 18 April 2016, https://clinicaltrials.gov/ct2/show/NCT02741596.

## Introduction

Hereditary angioedema (HAE) is a rare autosomal dominant disorder characterized by recurrent, unpredictable, and potentially life-threatening attacks of subcutaneous or submucosal swelling [1]. Attacks can be severely debilitating; patients with HAE are often limited in their ability to perform daily activities, and many experience anxiety and depression and poor health-related quality of life [2].

Lanadelumab is a fully human monoclonal antibody [3] that is approved in the United States, Europe, and several other countries/regions for the prevention of HAE attacks in patients at least 12 years old [4]. In the phase 3 HELP study (NCT02586805), attack rates were reduced by a mean of approximately 73–87% over 26 weeks’ treatment with lanadelumab (150 mg or 300 mg given every 2 or 4 weeks [q2w, q4w]) compared with placebo [5]. Subsequent evaluation of lanadelumab 300 mg q2w in the HELP open-label extension (OLE) study (NCT02741596) demonstrated the long-term benefit for patients with HAE, as the attack rate was reduced by a mean of approximately 87% over 132 weeks of treatment compared with baseline [6]. The safety profile in HELP OLE was consistent with that observed in HELP, with most treatment-emergent adverse events (TEAEs) being injection site reactions of mild severity.

HAE has a prevalence of 1 in 50,000 [7] and has been reported to affect all races [8]. However, the majority of patients included in clinical trials for HAE are White; thus minorities are vastly underrepresented, which may lead to racial and ethnic disparities in healthcare for patients with HAE [9]. Findings from an analysis of the effectiveness and safety of lanadelumab in subgroups of race and ethnicity in these 2 phase 3 clinical studies are presented herein. While the small number of patients in the minority groups precluded a statistical comparison, this analysis raises awareness of the need for greater representation of minority patients with HAE in future clinical trials.

## Methods

The HELP and HELP OLE studies enrolled patients aged ≥ 12 years with HAE type 1 or 2. Details of the studies have been reported previously [5, 6]. Briefly, in the randomized, double-blind, placebo-controlled HELP study, patients had a baseline rate of ≥ 1 investigator-confirmed attack in 4 weeks. They received placebo or lanadelumab (150 mg q4w, 300 mg q4w, or 300 mg q2w) for 26 weeks. The open-label HELP OLE study included patients who had completed HELP (rollovers) as well as patients who did not previously participate in HELP (non-rollovers); non-rollover patients had a historical baseline attack rate of ≥ 1 in 12 weeks. In the HELP OLE, patients received lanadelumab 300 mg q2w for up to 132 weeks. For both studies in this analysis, HAE attack rates during treatment were compared with baseline. Due to the small sizes of the minority groups, the comparisons were descriptive and a formal statistical analysis was not conducted. Similarly, the adverse event (AE) data were compared descriptively. Data from the three lanadelumab treatment groups in HELP were grouped together and outcomes were analyzed by race (White, Black/African American, Asian, and other) and by ethnicity (Hispanic/Latino and non-Hispanic/non-Latino).

## Results

### Patients

The HELP study enrolled a total of 125 patients, of whom 84 received lanadelumab. The HELP OLE study enrolled a total of 212 patients (109 rollover and 103 non-rollover) who received lanadelumab. In both studies, the majority of patients were White (HELP n = 74 [88.1%] and HELP OLE n = 198 [93.4%]) and non-Hispanic/non-Latino (HELP n = 77 [92.0%] and HELP OLE n = 198 [93.4%]). Most demographic and baseline disease characteristics were proportionally similar across the race and ethnicity groups; however, all racial and ethnic minority patients that were recruited were based at sites in the United States (Table 1).

**Table 1:** Baseline characteristics and demographics for patients in the lanadelumab groups in HELP and HELP OLE

Characteristic, n (%) unless otherwise stated	All lanadelumab groups (total)					
	Race				Ethnicity	
HELP (n = 84)^a^	White (n = 74)	Black/African American (n = 8)	Asian (n = 2)	Other (NA)	Non-Hispanic/non-Latino (n = 77)	Hispanic/Latino (n = 6)
Geographical region						
United States	51 (68.9)	8 (100)	2 (100)	–	54 (70.1)	6 (100)
Canada	4 (5.4)	0	0	–	4 (5.2)	0
Europe	17 (23.0)	0	0	–	17 (22.1)	0
Jordan	2 (2.7)	0	0	–	2 (2.6)	0
Race	–	–	–			
White				–	68 (88.3)	5 (83.3)
Black/African American				–	7 (9.1)	1 (16.7)
Asian				–	2 (2.6)	0
Ethnicity					–	–
Non-Hispanic/non-Latino	68 (91.9)	7 (87.5)	2 (100)	–		
Hispanic/Latino	5 (6.8)	1 (12.5)	0	–		
Unknown	1 (1.4)	0	0	–		
BMI, mean (SD)	28.8 (6.3)	28.2 (6.1)	25.8 (1.1)	–	28.7 (6.1)	29.4 (7.8)
Baseline HAE attack rate, mean (SD), attacks per month^b^	3.67 (2.25)	2.03 (1.51)	2.41 (1.99)	–	3.50 (2.20)	3.70 (2.69)
Lanadelumab treatment group in HELP						
150 mg q4w	25 (33.8)	1 (12.5)	2 (100)	–	27 (35.1)	1 (16.7)
300 mg q4w	23 (31.1)	6 (75.0)	0	–	27 (35.1)	2 (33.3)
300 mg q2w	26 (35.1)	1 (12.5)	0	–	23 (29.9)	3 (50.0)

Characteristic, n (%) unless otherwise stated	Lanadelumab 300 mg q2w					
	Race				Ethnicity	
HELP OLE (n = 212)	White (n = 198)	Black/African American (n = 10)	Asian (n = 2)	Other (n = 2)	Non-Hispanic/non-Latino (n = 198)	Hispanic/Latino (n = 13)
Geographical region						
United States	133 (67.2)	10 (100)	2 (100)	2 (100)	133 (67.2)	13 (100)
Canada	13 (6.6)	0	0	0	13 (6.6)	0
Europe	39 (19.7)	0	0	0	39 (19.7)	0
Jordan	13 (6.6)	0	0	0	13 (6.6)	0
Race	–	–	–	–		
White					185 (93.4)	12 (92.3)
Black/African American					9 (4.5)	1 (7.7)
Asian					0	0
Other					2 (1.0)	0
Ethnicity					–	–
Non-Hispanic/non-Latino	185 (93.4)	1 (10)	2 (100)	2 (100)		
Hispanic/Latino	12 (6.1)	9 (90)	0	0		
Unknown	1 (0.5)	0	0	0		
BMI, mean (SD)	28.3 (7.2)	28.4 (7.2)	25.8 (1.1)	31.1 (12.1)	28.3 (7.1)	28.5 (8.7)
Baseline attack rate, mean (SD), attacks per month^b^	3.13 (2.70)	1.82 (1.61)	2.41 (1.99)	1.38 (0.65)	3.02 (2.66)	3.37 (2.72)
Patient group in OLE						
Rollover	99 (50.0)	8 (80.0)	2 (100)	0	101 (51.0)	8 (61.5)
Non-rollover	99 (50.0)	2 (20.0)	0	2 (100)	97 (49.0)	5 (38.5)

*BMI* body mass index, *HAE* hereditary angioedema, *NA* not applicable, *OLE* open-label extension

^a^Of the 125 patients enrolled in the HELP study, 84 were enrolled to the lanadelumab cohorts and baseline characteristics for only these patients are reported here

^b^The baseline HAE attack rate was calculated for each patient as the number of investigator-confirmed HAE attacks that occurred during the run-in period of the HELP study for rollover patients or during the historical reporting period for non-rollover patients, divided by the number of days the patient contributed to the run-in period for rollover patients or historical reporting period for non-rollover patients, multiplied by 28 days. For non-rollover patients, the historical rate in the last 3 months before screening was used

### Efficacy and safety

Lanadelumab was effective in preventing HAE attacks across race and ethnicity groups in both the HELP and HELP OLE studies as indicated by a decrease in monthly attack rates (Table 2). In HELP, lanadelumab reduced mean attack rates from 3.67 attacks/month at baseline to 0.43 (88.2% reduction) in White patients (n = 74) and from 2.03 attacks/month to 0.48 (79.0% reduction) in Black/African American patients (n = 8). Of note, only 1 Black/African American patient was in the lanadelumab 300 mg q2w treatment group, which could account for the apparent lower extent of reduction compared to White patients. A mean attack rate reduction of 84.1% and 92.5% was observed in non-Hispanic/non-Latino (n = 77) and Hispanic/Latino patients (n = 6), respectively. Similar results were obtained in the HELP OLE; importantly, the mean (SD) attack rate during the treatment period was similar between White (n = 198) and Black/African American (n = 10) patients, and between Hispanic/Latino (n = 13) and non-Hispanic/non-Latino (n = 198) patients. Mean attack rates also decreased with lanadelumab treatment in the Asian (n = 2) and other (n = 2) patient groups; however, both of the Asian patients had been randomly assigned to receive a lower lanadelumab dose of 150 mg q4w.

**Table 2:** Monthly HAE attack rate reduction from baseline with lanadelumab treatment in HELP and HELP OLE

	Mean (SD) attack rate, attacks/4 weeks		Mean (SD) % change
	Baseline	Treatment period	
HELP			
White (n = 74)	3.67 (2.25)	0.43 (0.66)	− 88.18 (17.18)
Black/African American (n = 8)^a^	2.03 (1.51)	0.48 (0.44)	− 79.03 (19.42)
Asian (n = 2)^b^	2.41 (1.99)	1.74 (0.11)	12.61 (97.59)^c^
Non-Hispanic/non-Latino (n = 77)	3.50 (2.20)	0.49 (0.68)	− 84.11 (26.25)
Hispanic/Latino (n = 6)	3.70 (2.69)	0.31 (0.51)	− 92.53 (12.68)
HELP OLE			
White (n = 198)	3.13 (2.70)	0.25 (0.56)	− 87.74 (69.44)
Black/African American (n = 10)	1.82 (1.61)	0.26 (0.42)	− 78.94 (34.04)
Asian (n = 2)	2.41 (1.99)	0.34 (0.14)	− 75.10 (26.37)
Other (n = 2)	1.38 (0.65)	0.03 (0.04)	− 98.56 (2.04)
Non-Hispanic/non-Latino (n = 198)	3.02 (2.66)	0.26 (0.57)	− 86.60 (70.11)
Hispanic/Latino (n = 13)	3.37 (2.72)	0.06 (0.09)	− 97.66 (3.89)

Attack rates for the HELP study are for the lanadelumab treatment groups together (placebo is excluded). The data for all lanadelumab doses in the HELP study are pooled

*HAE* hereditary angioedema, *OLE* open-label extension, *q2w* every 2 weeks, *q4w* every 4 weeks

^a^Only 1 Black/African American patient was in the lanadelumab 300 mg q2w treatment group

^b^Both of the Asian patients were in the lanadelumab 150 mg q4w treatment group

^c^One patient had an increase in attack rate with treatment

**Fig. 1:**
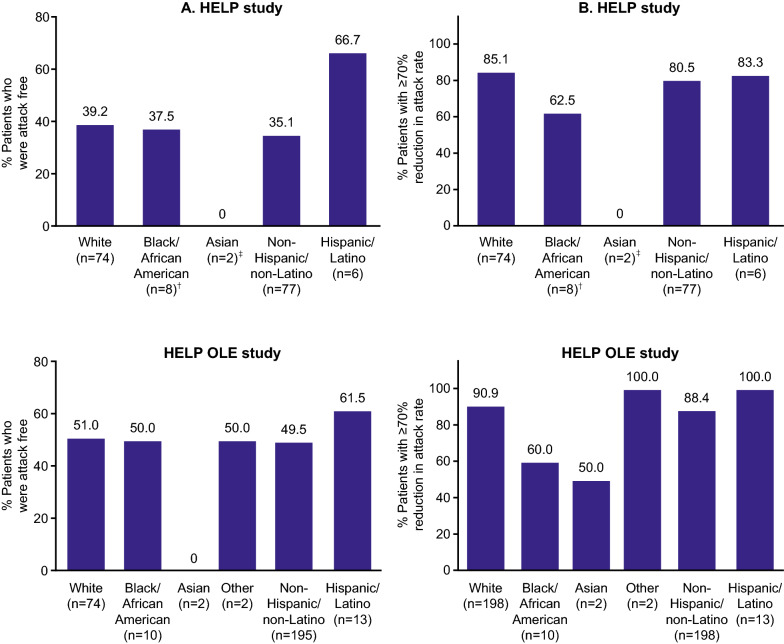
**A** Patients attack free 0–6 months after treatment initiation* and **B** with ≥ 70% attack rate reduction during treatment.* Data for all lanadelumab doses in the HELP study are pooled. *OLE* open-label extension, *q2w* every 2 weeks, *q4w* every 4 weeks. *Regular dosing stage for rollover patients in the HELP OLE study. ^†^Only 1 Black/African American patient was in the lanadelumab 300 mg q2w treatment group. ^‡^Both Asian patients were in the lanadelumab 150 mg q4w treatment group

A large proportion of patients across race and ethnicity groups were attack free from 0 to 6 months after initiation of lanadelumab treatment in both HELP and HELP OLE (Fig. 1). The proportion was highest for Hispanic/Latino patients in both studies: n = 4 (66.7%) Hispanic/Latino patients in HELP and n = 8 (61.5%) in HELP OLE were attack free, compared with n = 27 (35.1%) non-Hispanic/non-Latino patients in HELP and n = 98 (49.5%) in HELP OLE. In addition, n = 5 (62.5%) Black/African American patients and n = 63 (85.1%) White patients had an attack rate reduction of ≥ 70% during HELP. Similar proportions were observed in HELP OLE (n = 6 [60.0%] Black/African American and n = 180 [90.9%] White). This difference could be attributed to the lower baseline attack rate in Black/African American patients (2.03 attacks/month in HELP and 1.82 attacks/month in HELP OLE) compared with White patients (3.67 attacks/month in HELP and 3.13 attacks/month in HELP OLE).

The incidence of TEAEs was similar across race and ethnicity groups in both studies (Table 3). The most frequently reported TEAEs across all race and ethnicity groups in both HELP and HELP OLE were injection site pain and viral upper respiratory tract infection. Most TEAEs were mild to moderate in severity; there were no treatment-related serious TEAEs. Approximately half of the TEAEs were considered to be treatment related; injection site pain was the most commonly reported, and most of the reactions were mild.

**Table 3:** TEAEs reported in the HELP and HELP OLE studies by race/ethnicity

Adverse events, n (%)	All lanadelumab groups (total)					
	Race				Ethnicity	
HELP (n = 84)	White (n = 74)	Black/African American (n = 8)	Asian (n = 2)	Other (NA)	Non-Hispanic/non-Latino (n = 77)	Hispanic/Latino (n = 6)
Any TEAE	68 (91.9)	7 (87.5)	1 (50.0)	–	69 (89.6)	6 (100.0)
Most frequently reported TEAEs (in ≥ 5% of White or non-Hispanic/non-Latino groups)						
Injection site pain	30 (40.5)	5 (62.5)	1 (50.0)	–	34 (44.2)	2 (33.3)
Viral upper respiratory tract	19 (25.7)	1 (12.5)	0	–	18 (23.4)	1 (16.7)
Headache	14 (18.9)	3 (37.5)	0	–	15 (19.5)	2 (33.3)
Injection site erythema	8 (10.8)	0	0	–	8 (10.4)	0
Injection site bruising	6 (8.1)	0	0	–	6 (7.8)	0
Dizziness	5 (6.8)	0	0	–	5 (6.5)	0
Abdominal pain	4 (5.4)	0	0	–	2 (2.6)	0
Respiratory tract infection	4 (5.4)	0	1 (50.0)	–	1 (1.3)	1 (16.7)
Urinary tract infection	4 (5.4)	0	0	–	3 (3.9)	1 (16.7)
Myalgia	4 (5.4)	0	0	–	3 (3.9)	1 (16.7)
Rash	3 (4.1)	1 (12.5)	0	–	4 (5.2)	0
Toothache	0	0	0	–	4 (5.2)	0
Any treatment-related TEAE	42 (56.8)	7 (87.5)	1 (50.0)	–	47 (61.0)	3 (50.0)
Most frequently reported treatment-related AEs (in ≥ 5% of White or non-Hispanic/non-Latino)						
Injection site pain	29 (39.2)	5 (62.5)	1 (50.0)	–	33 (42.9)	1 (16.7)
Injection site erythema	8 (10.8)	0	0	–	8 (10.4)	0
Injection site bruising	5 (6.8)	0	0	–	5 (6.5)	0
Headache	4 (5.4)	2 (25.0)	0	–	4 (5.2)	2 (33.3)
Any serious AE	4 (5.4)	0	0	–	4 (5.2)	0
Any treatment-related serious AE	0	0	0	–	0	0
Any AE leading to discontinuation	1 (1.4)	0	0	–	1 (1.3)	0

Adverse events, n (%)	Lanadelumab 300 mg q2w					
	Race				Ethnicity	
HELP OLE (n = 212)	White (n = 198)	Black/African American (n = 10)	Asian (n = 2)	Other (n = 2)	Non-Hispanic/non-Latino (n = 198)	Hispanic/Latino (n = 13)
Any TEAE	194 (98.0)	8 (80)	2 (100)	2 (100)	192 (97.0)	13 (100.0)
Most frequently reported TEAEs (in ≥ 10% of White or non-Hispanic/non-Latino groups)						
Injection site pain	94 (47.5)	4 (40)	1 (50)	1 (50)	97 (49.0)	3 (23.1)
Viral upper respiratory tract infection	87 (43.9)	1 (10)	1 (50)	0	86 (40.3)	3 (23.1)
Upper respiratory tract infection	54 (27.3)	1 (10)	0	0	51 (25.8)	3 (23.1)
Headache	48 (24.2)	1 (10)	1 (50)	2 (100)	52 (26.3)	0
Injection site erythema	36 (18.2)	0	0	0	36 (18.2)	0
Injection site bruising	26 (13.1)	0	0	0	25 (12.6)	1 (7.7)
Arthralgia	25 (12.6)	2 (20)	0	0	26 (13.1)	0
Back pain	24 (12.1)	0	0	0	26 (13.1)	0
Diarrhea	23 (11.6)	0	0	0	23 (11.6)	0
Nausea	22 (11.1)	0	0	0	21 (10.6)	1 (7.7)
Sinusitis	22 (11.1)	1 (10)	0	0	23 (11.6)	0
Abdominal pain	21 (10.6)	1 (10)	0	0	22 (11.1)	0
Influenza	21 (10.6)	1 (10)	0	0	21 (10.6)	0
Urinary tract infection	21 (10.6)	2 (20)	0	0	20 (10.1)	0
Pain in extremity	20 (10.1)	0	0	0	20 (10.1)	1 (7.7)
Fatigue	19 (9.6)	1 (10)	0	0	20 (10.1)	0
Any treatment-related TEAE	109 (55.1)	5 (50)	1 (50)	1 (50.0)	112 (56.6)	3 (23.1)
Most frequently reported treatment-related AEs (in ≥ 5% of White or non-Hispanic/non-Latino)						
Injection site pain	75 (37.9)	4 (40.0)	1 (50.0)	1 (50.0)	80 (40.4)	1 (7.7)
Injection site erythema	29 (14.6)	0	0	0	29 (14.6)	0
Injection site bruising	15 (7.6)	0	0	0	14 (7.1)	1 (7.7)
Injection site swelling	10 (5.1)	0	0	0	10 (5.1)	0
Any serious AE	18 (9.1)	2 (20)	0 (0)		19 (9.6)	2 (15.4)
Any treatment-related serious AE	0 (0)	0 (0)	0 (0)		0 (0)	0 (0)
Any AE leading to discontinuation	6 (3)	0 (0)	0 (0)		4 (2.0)	1 (7.7)

*AE* adverse event, *TEAE* treatment-emergent adverse event, *NA* not applicable, *OLE* open-label extension; *q2w* every 2 weeks

## Discussion

To our knowledge, this is the first evaluation of the efficacy and safety of a specific treatment for HAE by race or ethnicity. In this assessment of data from the HELP and HELP OLE studies, lanadelumab was effective for the prevention of attacks in patients with HAE regardless of race or ethnicity, as indicated by reductions in monthly attack rates, the proportion of patients who were attack free in the first 6 months after treatment initiation, and the proportion of patients with a ≥ 70% reduction in attack rates from baseline. The safety profile of lanadelumab, as indicated by reported adverse events, was similar for all race and ethnicity groups.

Although there appear to be some differences in attack rate reduction between White and Black/African American patients, the minority patient groups in both studies were small; thus these findings are observational and comparisons between groups cannot be made. The percentage attack rate reduction and proportion of attack-free patients was lower in Black/African American patients compared with White patients, but it should be noted that the baseline attack rate was also lower in Black/African American patients, while the resulting attack rate during treatment with lanadelumab was similar to that in White patients. In addition, most Black/African American patients received a dose of 300 mg q4w and only one was in the highest dose group of 300 mg q2w in HELP, which may have contributed to the apparent differences between the groups.

Apparent differences in efficacy and safety for Asian patients could also be attributed to the very low number of Asian patients enrolled, as well as the administration in HELP of the lowest dosing regimen (150 mg q4w) studied. A study of lanadelumab efficacy and safety in Japanese patients (NCT04180163) [10] was recently completed and the findings will help to elucidate any differences in the response to lanadelumab in this population.

Similarly, the reduction in attack rate and proportion of attack-free patients was higher in Hispanic/Latino patients compared with non-Hispanic/non-Latino patients. Again, interpretation of these findings is difficult due to the small number of Hispanic/Latino patients enrolled.

Further evaluations in diverse populations are warranted given the small number of patients in each minority group. Historically, minorities are under-represented in clinical trials, and this is even more apparent in rare diseases such as HAE where the majority of clinical trial sites are located in North America or Europe; as such, more than 90% of trial patients identified as White race [11, 12], although a recent phase 2 trial included study sites in Asia [13]. HAE is a genetic disorder that has been reported in all races, with varying prevalence. An analysis of the GE Healthcare Centricity electronic medical records database covering 2006–2017 showed a prevalence of HAE in White patients of 76.8%, versus 8.3% in Black patients, 0.8% in Native American/Pacific Islander patients, and 0.4% in Asian patients [14]. Of note, the majority of confirmed HAE patients in the database were managed by a primary care physician; thus patients who were managed by an allergist may have been underrepresented. A recent retrospective study of data from the TriNetX Diamond Network covering more than 2000 patients with HAE from 2014 to 2021 showed an equal prevalence of HAE in White and Black populations (1.64 and 1.47 patients, respectively, per 100,000) and a lower prevalence in Hispanic patients (0.80 per 100,000) [15]. Underdiagnosis of rare genetic disorders in racial and ethnic minority groups is not uncommon [16–18] and may contribute to the apparent lower prevalence of HAE in these populations.

The broad classification of racial and ethnic groups (White, Black, Asian) may obscure differences in treatment impact between subgroups; for example, the prevalence of cardiovascular conditions was found to differ between Asian ethnic groups and versus an aggregate Asian group in a US study of electronic health records [19]. Additionally, health outcomes among Middle Eastern and North African (MENA) patients have been reported to differ from others categorized as White [20]. In the current analysis, patients from Jordan were classified as White at the time of enrollment, as MENA was not a standard clinical trial race category. It is thus important to identify any differences in clinical characteristics due to race and ethnicity, and subsequently any potential differences in treatment outcomes. Requirements for local clinical trials prior to drug registration in countries such as Japan, China, and India can provide an opportunity to obtain such data, but these may not be sufficiently inclusive for the diverse global HAE patient population.

HAE is a highly heterogenous disease, but only a few studies have indicated differences in presentation between populations. Speletas et al. suggested differences in the prevalence of specific mutations in *SERPING1* (the gene that encodes C1-inhibitor, which is deficient in HAE) in various European populations, which may lead to differences in disease severity [21]. In addition, a retrospective Chinese study reported a lower frequency of abdominal attacks in patients with HAE in China, Taiwan, and Japan than has been reported for patients in Western countries [22]. The frequency of attacks affecting the gastrointestinal system also appeared to be low among patients in South Korea [23] and Japan [24].

Notably, there was a disparity in the geographical location of patients recruited to these studies, in that the racial and ethnic minority groups that were recruited were unintentionally based only in sites in the United States, which may have had a confounding effect on the results. It is well known that disparities exist not only in the medical care received by minority Americans, but differences in the responses to pharmaceutical treatments of these groups have been insufficiently acknowledged [25, 26]. For example, certain anti-hypertensive medications are less effective in Blacks compared to Whites [27]. In addition, racial variation in adverse effects has been well demonstrated [28, 29]. Notably, adverse reactions to anti-convulsive medications vary considerably between Whites and Asians with Vietnamese ancestry [30], emphasizing the need to investigate drug efficacy in minorities. Beyond our study there are limited data on diagnosis and treatment options for racial and ethnic minorities with HAE; however, evidence suggests there is a need for improvement. In a cross-sectional study evaluating quality of life for Hispanic patients with HAE in Puerto Rico, significantly lower scores were reported in both the physical and mental components of the Short Form-36v2 questionnaire than the US population norms [31]. Furthermore, a higher frequency of attacks was reported in the Puerto Rican population despite a similar epidemiologic and clinical profile to previous studies [31]. A report from the first Latin American HAE Patient Advocacy Forum held in 2013 highlighted that HAE is highly underrecognized and undertreated in that region [32]. Limited knowledge of the condition, diagnostic difficulties, and the insufficient availability of effective treatments or prophylaxis options highlight the vulnerability for the Latin American HAE patient community [32]. These findings confirm that there is a potentially significant knowledge gap for healthcare providers in diagnosis and treatment of minority patients with HAE.

In conclusion, the efficacy and safety of lanadelumab were similar regardless of race and ethnicity in the HELP and the HELP OLE studies, but greater knowledge of diagnosis and treatment of HAE for these minorities is required to ensure adequate care of minorities. Efforts should be made to increase the recruitment of minority patients into studies, regardless of the difficulties of accomplishing this in orphan diseases secondary to small patient numbers. The safety and efficacy of medications should be ensured in all races and ethnicities.

## Data Availability

The redacted study protocol and redacted statistical analysis plan for the HELP study have been previously published [5]. The redacted study protocol and redacted statistical analysis plan for the HELP OLE study, as well as the data sets, including individual participants’ data supporting the results of HELP and HELP OLE, will be made available after the publication of study results within 3 months from initial request to researchers who provide a methodologically sound proposal. The data will be provided after its de-identification, in compliance with applicable privacy laws, data protection, and requirements for consent and anonymization.

## Abbreviations

AE: Adverse event
HAE: Hereditary angioedema
OLE: Open label extension
TEAE: Treatment-emergent adverse event
